# Unraveling Glioblastoma Heterogeneity: Advancing Immunological Insights and Therapeutic Innovations

**DOI:** 10.3390/brainsci15080833

**Published:** 2025-08-02

**Authors:** Joshua H. Liu, Maksym Horiachok, Santosh Guru, Cecile L. Maire

**Affiliations:** 1 Department of Medicine, Washington University School of Medicine, St. Louis, MO 63110, USA; joshualiu@wustl.edu; 2 Medical Faculty, Bukovinian State Medical University, 58002 Chernivtsi, Ukraine; maxgor@bsmu.edu.ua; 3 School of Clinical Medicine, University of Cambridge, Cambridge CB2 1TN, UK; sg928@cam.ac.uk; 4 Department of Neurosurgery, University Medical Center Hamburg-Eppendorf, 20251 Hamburg, Germany

**Keywords:** glioblastoma, heterogeneity, immunosuppression, T cell exhaustion

## Abstract

Glioblastoma (GBM) remains one of the most aggressive and treatment-resistant brain tumors, largely due to its profound intratumoral heterogeneity and immunosuppressive microenvironment. Various classifications of GBM subtypes were created based on transcriptional and methylation profiles. This effort, followed by the development of new technology such as single-nuclei sequencing (snRNAseq) and spatial transcriptomics, led to a better understanding of the glioma cells’ plasticity and their ability to transition between diverse cellular states. GBM cells can mimic neurodevelopmental programs to resemble oligodendrocyte or neural progenitor behavior and hitchhike the local neuronal network to support their growth. The tumor microenvironment, especially under hypoxic conditions, drives the tumor cell clonal selection, which then reshapes the immune cells’ functions. These adaptations contribute to immune evasion by progressively disabling T cell and myeloid cell functions, ultimately establishing a highly immunosuppressive tumor milieu. This complex and metabolically constrained environment poses a major barrier to effective antitumor immunity and limits the success of conventional therapies. Understanding the dynamic interactions between glioma cells and their microenvironment is essential for the development of more effective immunotherapies and rational combination strategies aimed at overcoming resistance and improving patient outcomes.

## 1. Introduction

Glioblastoma (GBM) is the most common and lethal primary malignant brain tumor in adults, accounting for approximately 50% of all primary central nervous system (CNS) malignancies and occurring at an annual incidence of ~3 cases per 100,000 individuals [1]. Standard-of-care treatment, comprising maximal surgical resection followed by concurrent chemoradiotherapy with temozolomide, offers only modest benefits, with a median overall survival ranging from 12 to 18 months and a five-year survival rate below 10% [2]. These outcomes highlight the urgent need for innovative therapies capable of overcoming GBM’s distinct biological and immunological barriers.

Immunotherapy has emerged as a powerful modality across multiple cancer types, particularly in tumors such as melanoma and non-small cell lung cancer, where immune checkpoint inhibitors and T cell-based therapies have produced durable clinical responses [3]. These successes have spurred significant interest in developing immunotherapeutic strategies for GBM. However, clinical trials of immune checkpoint blockade, cancer vaccines, and adoptive T cell therapies have largely failed to demonstrate survival benefit in patients with GBM [4]. This discrepancy reflects the unique immunological landscape of GBM, characterized by profound immunosuppression, limited T-cell infiltration, and the reduced expression of tumor antigens, challenging the implementation of immunotherapies in GBM [5]. Glioma cells actively suppress immune surveillance through multiple mechanisms, including the downregulation of MHC molecules, recruitment of regulatory immune populations, expression of immune checkpoint ligands, and secretion of immunosuppressive cytokines [6]. Moreover, the presence of the blood–brain barrier further limits immune cell trafficking and therapeutic delivery. As a result, tumor-infiltrating T cells in GBM are often functionally exhausted and numerically sparse, diminishing their ability to mediate effective antitumor responses [7].

In addition to the deleterious immunosuppressive microenvironment, the remarkable intratumoral heterogeneity and complexity of glioma cell growth pose major challenges for effective therapy design. Over the past two decades, efforts to refine glioma classification have led to a new definition of glioma stem cells and uncovered the extraordinary plasticity of GBM cells in adapting to their environment. Advances in transcriptomic and epigenetic profiling, including single-cell RNA sequencing, have revealed that glioma cells can mimic multiple neurodevelopmental states [8]. These diverse cellular identities—often coexisting within a single tumor—contribute to therapy resistance and tumor progression. Moreover, spatial and temporal analyses have shown that glioma evolution is driven by clonal dynamics shaped by both genetic and epigenetic alterations.

Despite these challenges, T cells remain a central focus of GBM immunotherapy. Recent efforts aim to enhance T-cell priming, trafficking, and persistence within the GBM microenvironment through strategies such as antigen-targeted vaccines, chimeric antigen receptor (CAR) T cells, bispecific T cell engagers, and combination checkpoint blockade [9]. Furthermore, insights into T-cell dysfunction, exclusion, and exhaustion in GBM are informing the development and refinement of immunotherapies that seek to reprogram the immune landscape of the disease. Together, these advances underscore the continued premise of T cell-based immunotherapy treatments in GBM, while emphasizing the need for a deeper mechanistic understanding of immune evasion and tumor cell heterogeneity in this uniquely challenging malignancy.

## 2. Tumor Cell Heterogeneity

Over the past two decades, extensive research efforts have been devoted to defining and characterizing tumor cells that comprise GBM. It rapidly became evident that the tumor cells’ heterogeneity, driven by genetic alterations such as copy number variation and point mutation, required a subclassification of the GBM. Early studies using bulk RNAseq and FISH first defined three to four new GBM subtypes based on major pathway activation, which ultimately led to the discovery of IDH-mutant glioma as a distinct entity [10,11,12,13,14,15]. Subsequent advances in machine learning-based classifiers and DNA-methylation-based arrays gave rise to a new classification of the GBM in three subclasses (RTK1, RTK2, and MES) [16]. In parallel with epigenetic classification, transcriptomic profiling has gained considerable depth and precision with the integration of single-cell RNA sequencing (scRNAseq) analyses. These studies have demonstrated that the canonical transcriptomic subtypes of proneural, classical, and mesenchymal could co-exist within a single tumor [17]. Furthermore, longitudinal analysis of paired samples pre- and post-standard of care samples has highlighted treatment-induced changes, with a subset of recurrent tumors exhibiting characteristic temozolomide-associated mutagenesis. This suggests that treatment with alkylating agents triggers specific mutations in glioma cells, potentially rendering these hypermutated tumors more sensitive to immune checkpoint inhibitors [18,19,20].

The initial concept that a subset of tumor cells in GBM possesses stem cell–like properties, such as slow proliferation and the capacity to regenerate the entire tumor, positioning them as key drivers of resistance to chemo- and radiotherapy and as ideal therapeutic targets, has evolved into a more nuanced understanding of tumor cell plasticity, largely informed by insights from scRNAseq [21,22]. These analyses led to the identification of four distinct cellular states: neural progenitor cell (NPC)-like, astrocyte (AC)-like, oligodendrocyte progenitor cell (OPC)-like, and mesenchymal (MES)-like, introducing the concept that GBM tumor cells can mimic multiple stages of neuronal development [23]. Interestingly, this work also demonstrates that these cellular states can coexist within a single tumor, with their distribution varying according to copy number alterations. For example, the transcriptionally defined proneural subtype is composed predominantly of a mixture of NPC and OPC-like cells. Analysis of the rare and self-renewing glioma stem cells population shows a previously unknown diversity and molecular heterogeneity with transcriptional signatures defining two cell states around Developmental and Injury Response programs [24,25]. The notion that glioma cells, in diverse cellular states, mimic neurodevelopmental trajectories has also been corroborated at the epigenetic level, with DNA methylation changes reflecting the cellular state and contributing to their progression [26,27].

Ten new groups representing malignant tumor cell states, based on their correlation with normal cell development or activities such as proliferation or hypoxia, were recently described through single-nucleus RNA-seq analysis of a large cohort of primary and recurrent GBM [28,29]. Each group (OPC-like, AC-like, hypoxia, MES-like, NPC-like, GPC-like, NEU-like, stress, cilia, and cycling) defines a distinct cell state that can be linked to spatial transcriptomic data to characterize tumor ecosystems. These analyses revealed a tumor architecture far more complex than previously described using classical histological techniques, with cellular states extending beyond conventional histopathological borders. Additionally, certain tumor regions appear highly disorganized, whereas others exhibit a layered organization, with distinct microenvironments positioned adjacent to one another [30]. Epigenetic analysis through Hi-seq and single-nuclei ATAC-seq of specific tumor regions showed that GBM clonal expansion is driven by tumor suppressor deletions, which could occur independently across different regions of the tumor. Major genomic alterations, such as chromothripsis, disrupt the epigenetic code as very early events, driving the clonal evolution of the GBM. In some patient samples, chromatin and transcriptome analysis confirmed the neurodevelopmental lineage origin of GBMs, with activation of pioneer transcription factors such as NEUROD1 contributing to both GBM heterogeneity and the emergence of a primitive neuronal signature [31]. A specific subtype of glioma stem cell has recently been identified to closely resemble outer radial glia (oRG) cells, which have the capacity to give rise to transient amplifying cells that could differentiate into bipotential progenitor cells capable of regenerating brain cells in contexts of stroke or multiple sclerosis. These oRG-like glioma stem cells exhibit a high invasive potential driven by activation of the hub genes PTPRZ1, TNC, and LIFR [32].

Conceptual models of GBM heterogeneity and clonal evolution have thus shifted from the notion of a single dominant clone driving the tumor growth toward frameworks that incorporate spatial and temporal dynamics. Subtle selected advantages at the single-cell level, such as enhanced migratory capacity, resistance to hypoxic microenvironment, or the ability to adapt to new neighboring cell population, fuels tumor heterogeneity and clonal selection. Understanding these processes is increasingly recognized as critical for identifying new therapeutic targets and mitigating chemoresistance [33,34].

## 3. The Brain-Specific Tumor Microenvironment

**Figure 1:**
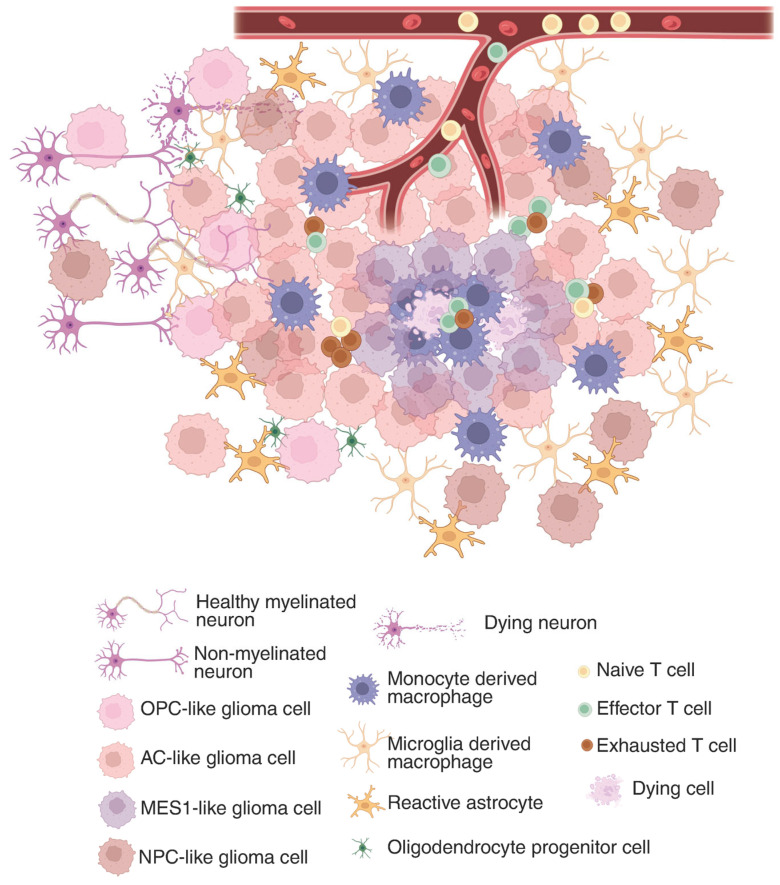
Glioma immunosuppressive landscape in the tumor microenvironment. In the tumor core, where a hypoxic environment is predominant, MES1-like glioma cells are sequestering tumor-associated macrophages and CD8^+^ T cells. While in the periphery, glioma cells state change to more OPC or NPC-like and hitchhike the neuronal network. The composition of the tumor microenvironment constantly affects the glioma cells’ functions and drives their clonal evolution, which shapes the immunosuppressive landscape. Created in BioRender. Maire, C. (2025) https://BioRender.com/z6cwosx (accessed 29 July 2025).

The GBM tumor microenvironment (TME) is a dynamic and complex system composed of all non-cancerous components within the tumor, such as immune cells (T cells, microglia/macrophages, dendritic cells, neutrophils), endothelial cells, and vascular pericytes, but also local neurons, astrocytes, and oligodendrocyte progenitors (Figure 1). In addition to these cellular elements, the TME contains diverse signaling molecules, including cytokines, chemokines, growth factors, hormones, nitric oxide (NO), and various polysaccharides [35]. Together, these constituents create a specialized ecosystem that supports tumor growth, facilitates immune evasion, and contributes substantially to treatment resistance.

GBM’s immunosuppressive TME plays a critical role in shielding the tumor from immune-mediated destruction. While some tumors elicit robust immune responses, GBM is often described as immunologically ‘cold’ due to the paucity of tumor-infiltrating T cells, contributing to its profoundly immunosuppressive milieu. Moreover, the blood–brain barrier, along with the potential immune-privilege nature of the CNS, limits antigen presentation and restricts lymphocyte infiltration, thereby reducing the effectiveness of immunotherapies [36].

### 3.1. Glioma Cells Adapt to Their Location: Periphery vs. Tumor Core

Infiltrating tumor cells, which preferentially migrate along white matter tracks, present the ability to interact with the normal brain cells, hitchhiking their neuronal networks to ensure their survival. As these cells migrate away from the hypoxic tumor core into more oxygen-rich regions of the brain, they downregulate genes associated with hypoxia adaptation. Additionally, activation of the Fibroblast Growth Factor (FGF) signaling pathway and enzymes involved in proline catabolism further fuel these infiltrating tumor cells, providing an ATP-rich metabolic state. Notably, these cells present a strong resemblance to the gene signature of oligodendrocyte progenitor cells and to the fetal human astrocyte gene signature [37]. In the peripheral region, infiltrating tumor cells gain the capacity to mimic oligodendrocyte development by upregulating transcription factors known to be essential for OPC maintenance, such as ZEB1, OLIG2, ASCL1, and TCF family. Interestingly, these tumor cells appear to maintain such a cellular state by interacting with healthy neurons along their migration path [38].

Remarkably, invading tumor cells also have the capacity to maintain such a specific cellular state and transcriptional signature after transplantation [39]. Beyond proliferation, the ability of these cells to establish synaptic activity and connect with neurons provides a critical advantage for infiltrating the normal brain parenchyma. Recent studies have highlighted the importance of the glioma–neuron interactions in this process, showing that the glioma cells are synaptically integrated to the normal neuronal circuitry and receive survival signals through neuronal secretion of BDNF and NLGN3. Excitatory glutamate neuro-gliomal synapse and BDNF binding to NTRK2 receptors on tumor cells induced expression of glutamatergic AMPA receptors, leading to strong glutamate signaling [40,41,42]. The high concentration of glutamate released by the glioma cells, however, leads to neuronal damage and death of the GABAergic inhibitory interneurons or the conversion into excitatory neurons due to the loss of their potassium/chloride transporter KCC2 [43]. Ultimately, the extent of connectivity between the glioma cells and neurons contributes to cognitive decline and correlates with poor survival [44]. In vivo multiphoton laser-scanning microscopy, allowing tracking of Ca^2+^ flux between tumor cells, showed that the glioma cells in the tumor core are interconnected. Glioma cells organize themselves into an extensive network through Connexin-43+ gap junctions on microtubules [45,46]. In certain GBM cases, these pacemaker-like cells display a rhythmic Ca^2+^ oscillation that drives synchronous activity within the tumor cell network, increasing the tumor aggressiveness through activation of the MAPK and NFkB pathway, leading to radio and chemotherapy resistance [47].

### 3.2. Niches in the TME

During the invasion of the normal brain tissue, glioma cells frequently migrated toward highly vascularized regions. They have the capacity to closely associate with blood vessel endothelial cells, forming satellite-like structures known as the perivascular niche [48]. Glioma cells that have the capacity to co-opt the brain vasculature adopt a specific functional state termed VC-Resist (vessel co-opting and resistant cell state), which shares transcriptomic similarities with the AC/MES-like state but displays a higher resistance to DNA damage. Combined with their ability to disrupt blood vessel permeability, these cells are a major contributor to radio and chemoresistance [49]. Leakage of the blood–brain barrier (BBB) and damage to the peripheral blood vessels result in inadequate oxygen delivery, triggering macrophages recruitment and exacerbating local environment dysregulation [50,51].

Hypoxia, a hallmark of GBM, plays a central role in shaping the organization of the tumor microenvironment. It extends beyond classical histopathological features and varies markedly across the tumor mass, ranging from the central necrotic core to scattered low-oxygen regions. This oxygen gradient induces a broad spectrum of cellular adaptations, with some regions undergoing apoptosis or necrosis while others activate survival mechanisms that support growth under metabolic stress. Spatial transcriptomic studies have revealed a distinct cellular architecture organized around hypoxic zones, characterized by mesenchymal (MES) like signatures with high expression of NDRG1 and CHI3L1, surrounded by immunosuppressive macrophages, aberrant vasculature, and glioma cells with neurodevelopmental functional state expressing OLIG2 and DCX [30]. Glioma cells adapt to hypoxic stress by modulating and expressing their energy sensor AMP-activated protein kinase (AMPK), which confers a metabolic advantage, enabling them to resist hypoxia-induced cell death and survive under glucose deprivation [52].

To better comprehend the functional and cellular organization of the hypoxic TME, a mouse model with UnaG, a fluorescent protein that does not require oxidation to become fluorescent, was created under the control of HIF response elements. The UnaG reporter reliably tracks HIF activity in hypoxic cells and confirmed the extent and distribution of hypoxia in GBM. Staining on human MES GBM validated the mouse findings by showing that hypoxic glioma cells secret IL1B and CCL8 to entrap GLUT1+ macrophages, a direct target of HIF, along with the recruitment of CD8^+^ cytotoxic [53], forming pseudopalisades structures. Additionally, the hypoxic signature defined by the UnaG+ cells could be matched to the human cohort, which was associated with poor survival and was predominantly found in recurrent tumors at specific locations such as the perinecrotic zone and pseudopalisading cells [54].

Collectively, studies investigating the hypoxic microenvironment in GBM increasingly suggest that the MES subtype likely represents a tumor microenvironment-driven signature rather than a glioma cell intrinsic cellular state. Moreover, macrophages appear to be the drivers of the MES phenotype and are also implicated in the upregulation of cytotoxic T-cell programs [30,55].

### 3.3. Myeloid Cells in the GBM Tumor Microenvironment

In numerous tumor types, myeloid cell populations become elevated and markedly dysregulated both systemically and within the TME, associated to poor prognosis [56,57]. In glioma, high expression of myeloid lineage markers has been associated with profound reshaping of the immune landscape compared with healthy brain tissue [58]. Tumor-associated macrophages (TAMs) constitute the predominant immune cell population within the GBM TME, comprising up to 50% of immune cells, whereas the lymphocyte population accounts for only 2% [59]. The relative contributions of bone marrow-derived myeloid cells versus resident microglia in the TAM population remain uncertain in human GBM and cannot be extrapolated from the mouse models [60]. The type of brain tumor, namely glioma with IDH wild-type, IDH mutation, or brain metastasis (BrM), clearly influences both the composition of TAMs and the degree of immunosuppression. RNAseq combined with flow cytometry analyses revealed that GBM, as well as certain BrMet, preferentially recruit monocyte-derived macrophages rather than activating resident microglia [59]. Regardless of their origin, TAMs undergo profound alterations when in the presence of glioma tumor cells. Their activation state cannot be adequately described by the traditional M1-M2 classification [61,62]. Instead, TAMs exhibit highly diverse transcriptional programs, including a signature of type I interferon and IL-4 signaling, underscoring their remarkable plasticity and heterogeneity [59]. Advanced technologies like snRNAseq and spatial transcriptomics have further demonstrated that, similar to GBM cells, TAMs’ functional states vary significantly according to their spatial localization within the tumor [63]. Microglia-derived macrophages are enriched at the tumor periphery, adjacent to OPC and NPC-like glioma cells. In contrast, macrophages within hypoxic regions and adjacent to MES1-like tumor cells display a scavenger, immunosuppressive signature characterized by the expression of CD206, CD204, and CD163, as well as immunosuppressive molecules such as NRP1, RNASE1, and CTSB, which are known to inhibit T cell proliferation [64,65]. The role of TAMs in driving T cell exhaustion in GBM has been recently investigated, revealing that TAMs–T cell interaction or TAMs selective depletion may represent more promising therapeutic strategies than immune checkpoint blockage alone, which has thus far shown limited efficacy in GBM [66].

Metabolic reprogramming also plays a key role in how TAMs shape the tumor immune landscape. GBM TAMs exhibit elevated cholesterol biosynthesis and accumulation, linked to the expression of anti-phagocytic molecules such as Siglec019 and PD-1 [67]. The HIF-1a-driven glycolytic environment also influences TAMs through upregulation of glutamine synthetase and glutamate transporters such as GluA2, EAAT1, EAAT2, induced by increased enzymatic activity of a-KG (a-ketoglutarate), resulting in elevated glutamate levels [68]. These adaptations not only enhance myeloid cell survival in the harsh TME but also reinforce the immunosuppressive nature of GBM.

Myeloid-derived suppressor cells (MDSCs) constitute a phenotypically diverse group of immature myeloid cells that lack the surface markers of mature lymphoid and myeloid cells, such as the MHC class II molecule, HLA-DR [69]. An increased amount of MDSCs, especially the neutrophilic type PMN-MDSC, has been found in the peripheral blood of GBM patients [70]. These cells induce immunosuppression via the production of reactive oxygen species (ROS) and nitrogen species (RNS). RNS activates ARG1 or iNOS, which depletes L-arginine and impairs expression of CD3ζ, thereby inhibiting T and NK cell proliferation [71,72]. Reduced arginine levels induce activation of polyamine synthesis, particularly putrescine, leading to an increased resistance of the MDSC to acidic TME, promoting immunosuppression even further [73].

## 4. T Cell Subtype Function and Presence in GBM TME

Although T lymphocytes represent a minority of infiltrating immune cells in GBM tissue, their presence significantly shapes the TME and broader immune landscape. T cells can both contribute to antitumor immunity or promote tumor progression, depending on their subtype, activation status, and interactions with the local microenvironment.

Multiple mechanisms facilitate the recruitment of T lymphocytes across the BBB and into GBM tissue, including BBB disruption and chemotactic signaling by tumor cells and TAMs. Once across the BBB and into the brain parenchyma, T cells are further recruited into tumor sites by chemokine-mediated cues. GBM tumor cells and associated myeloid populations produce immunomodulatory factors such as indoleamine 2,3-dioxygenase (IDO), CCL2, and CCL22, which contribute to the recruitment and regulation of T cell populations within the tumor microenvironment [74,75].

The GBM tumor microenvironment contains a heterogeneous mix of tissue-infiltrating lymphocytes (TILs) subtypes. The dynamic interplay between T cells and both neoplastic and non-neoplastic cells contributes to an ever-evolving immunological landscape where an equilibrium can be established between pro-tumoral T cells that increase tumor growth and anti-tumoral effector T cells that inhibit growth and kill tumor cells [76]. Broadly, these can be categorized into effector T cells, including cytotoxic CD8^+^ and helper CD4^+^ T cell subsets, and immunosuppressive regulatory T cells [77,78].

### 4.1. CD8^+^ Cytotoxic T Lymphocytes

CD8^+^ cytotoxic T lymphocytes (CTLs) play a central role in antitumor immunity by directly recognizing and eliminating tumor cells [79,80,81,82]. While relatively low in numbers in GBM, these cells are frequently detected within the TME. Tumor-related antigens can be recognized by CD8^+^ T cells in the context of major histocompatibility complex (MHC) class I-expressing tumors. The activation of CD8^+^ T cells is a tightly regulated process influenced by antigen characteristics, the local immunological environment, and the duration of antigen exposure. Under physiologic conditions, naïve CD8^+^ T cells become activated upon encountering a neoantigen presented by various myeloid antigen-presenting cells (APCs), leading to their clonal expansion and differentiation into effector CTLs capable of directly lysing target cells expressing the relevant antigen [83]. Once activated, CTLs survey the tumor microenvironment and eliminate target cells by recognizing specific antigens through their T-cell receptors and inducing apoptosis via cytotoxic granule release or engagement of death receptor pathways such as the Fas-FasL interaction [84].

However, in the context of GBM, this activation cascade can be disrupted within the tumor microenvironment. Tumor cells can evade immune recognition through impaired antigen processing or presentation, or by interfering with T cell priming. Such mechanisms contribute to dysfunctional T cell states that ultimately promote immune evasion and tumor progression. In the context of glioblastoma, several distinct dysfunctional states have been described, including senescence, tolerance, anergy, exhaustion, and ignorance, which will be discussed in later sections [85].

### 4.2. Effector CD4^+^ Cells

Among effector T cell populations, CD4^+^ helper T cells are also present within the glioblastoma microenvironment, though they remain less extensively characterized than their CD8^+^ counterparts. CD4^+^ T cells modulate antitumor immunity by secreting cytokines, activating antigen-presenting cells, and coordinating broader immune responses. Notably, effective CD8^+^ T cell responses often depend on sufficient CD4^+^ T cell help; in the absence of such support, CD8^+^ T cells frequently exhibit suboptimal activation and effector function. Within the tumor microenvironment, CD4^+^ T cell-derived cytokines such as interferon-gamma (IFN-γ) contribute to the recruitment and homing of immune cells to the tumor site, while interleukins such as IL-2 and IL-21 are essential for the differentiation and maintenance of high-affinity cytotoxic CD8^+^ T cells [79,86].

### 4.3. CD4^+^ Regulatory T Cells

However, the immunological role of CD4^+^ TILs in GBM is complex. While some subsets support antitumor immunity, others contribute to immune suppression. A subset of CD4^+^ T cells found within the GBM tumor microenvironment includes regulatory T cells (Tregs), characterized by the expression of CD25 and the transcription factor Foxp3, and are frequently enriched in the GBM microenvironment and are thought to suppress local immune activity, thereby facilitating tumor immune evasion. Under physiological conditions, Tregs play a crucial role in maintaining immune homeostasis by preventing autoimmunity and limiting excessive immune activation. However, in the context of GBM, their immunosuppressive function is co-opted, and an increased abundance of Tregs in both the TME and peripheral circulation has been observed, followed by diminished antitumor immunity and poor responsiveness to immunotherapy [87,88].

Interestingly, Tregs also exhibit distinct trafficking behavior, guided by chemokine receptor expression that facilitates their infiltration into the TME. Chemokine gradients—particularly involving receptors such as CCR4, CCR8, CCR10, and CXCR3—mediate Treg migration from the thymus to sites of inflammation or tumor. In GBM, tumor-derived chemokines such as CCL2 and CCL22 bind to CCR4 on Tregs and have been implicated in their selective recruitment to the tumor [88]. Notably, approximately 74% of Tregs isolated from the peripheral blood of GBM patients express CCR4, compared with only about 43% in healthy individuals, suggesting that GBM may induce or upregulate CCR4 expression on Tregs through soluble factors in the tumor milieu [89]. Beyond peripheral recruitment, Tregs may also be generated locally within the TME through the in situ conversion of naive CD4^+^ T cells. Tumor-secreted soluble factors, such as transforming growth factor-beta (TGF-β) and PD-L1, can induce FOXP3 expression in conventional CD4^+^ T cells, resulting in the generation of Tregs with immunosuppressive capabilities [74]. Although thymus-derived Tregs are believed to constitute the majority of Tregs within the TME [90], this local induction mechanism likely contributes to the elevated Treg burden observed in GBM. Supporting this, TGF-β and PD-L1 neutralization has been shown to reduce the number of tumor-infiltrating Tregs in murine models of brain tumors, implicating this pathway as a potential driver of Treg accumulation in the GBM microenvironment [91,92].

The proliferation of Treg cells is fueled by immunosuppressive cytokines like TGF-β and IL-10, produced by both tumor cells and resident immune populations [93]. Once within the TME, Treg cells exert immunosuppressive effects through multiple molecular mechanisms. Foxp3^+^ Tregs inhibit APC function by engaging CD80/CD86 via cytotoxic T-lymphocyte-associated protein 4 (CTLA-4), thereby reducing costimulatory signaling and impairing T cell activation. These cells also secrete immunosuppressive cytokines, including TGF-β, IL-10, and IL-35, as well as sequestering IL-2, collectively suppressing T cell proliferation and function. In addition, Tregs can induce apoptosis of effector T cells through the release of cytotoxic mediators such as perforin and granzyme [94]. Expression of immune checkpoint molecules, including CTLA-4, inducible T-cell co-stimulator (ICOS), and lymphocyte activation gene-3 (LAG-3), further contributes to the suppression of cytotoxic T cell function and proliferation, reinforcing the immunosuppressive milieu of the GBM TME. While the immunosuppressive pattern of Tregs has been extensively described, their prognostic impact in GBM remains unclear, with conflicting evidence across studies [95,96,97].

A distinct subset of Tregs, known as T follicular regulatory (Tfr) cells, co-expresses FoxP3 and Bcl-6 and expands clonally upon recognizing tumor neoantigens. It has been identified that Tfr cells exist across various tumors and remain transcriptionally divergent from conventional Tregs [98]. Moreover, Lu et al. detected increased Tfr and Treg populations in glioma samples, both of which suppressed CD8^+^ T cell proliferation. Beyond that, glioma-derived Tfr cells showed stronger immunosuppressive effects compared with those from peripheral blood. These findings underscore the therapeutic relevance of targeting Tfr and Treg cells in glioma [99].

### 4.4. Clinical Utility of T Cell Subtypes

The clinical use of infiltrating T cells has been well established in several malignancies, including melanoma, ovarian, breast, and colorectal cancers, where their presence is often associated with improved patient outcomes. However, in GBM, the prognostic and therapeutic implications of T cell infiltration remain less clear and more context-dependent. Early studies assessing total TIL density in GBM yielded mixed results. While some reported a positive correlation between TIL presence and overall survival, others found no significant association or even suggested a negative impact on prognosis [100,101,102]. These inconsistencies likely reflect the oversimplified treatment of TILs as a homogeneous population, without accounting for the functional diversity of T cell subtypes.

Recent efforts to better decipher and characterize the TIL populations have offered greater insight. Higher levels of CD8^+^ T cell infiltration, especially when coupled with signs of activation and proliferation, have been associated with improved survival in several GBM cohorts [81,82,103,104]. Similarly, increased infiltration of effector T cell subsets as a whole, including both cytotoxic and helper T cells, may reflect a more robust antitumor immune response and has correlated with favorable outcomes [105]. Conversely, the immunosuppressive activity of Tregs has been linked to tumor-mediated upregulation of enzymes such as indoleamine 2,3-dioxygenase 1 (IDO1), which may further contribute to immune evasion [106]. These findings highlight the importance of the balance between effector and suppressive T cell populations in shaping the tumor immune landscape.

From a translational standpoint, a more nuanced understanding of TIL composition could directly inform patient stratification for immunotherapy trials. For example, pre-treatment profiling of T cell subtypes such as CD8^+^/Treg ratios or expression of activation/exhaustion markers (PD-1, TIM-3, granzyme B) or T cell clonal diversity in the primary tumor, may help identify patients more likely to benefit from checkpoint blockade or adoptive cell therapies. These immune features may also serve as pharmacodynamic biomarkers to monitor response or resistance. Furthermore, the spatial organization of T cells within the TME (perivascular niche vs. intratumoral) could provide additional predictive value, particularly in the context of therapies targeting the TME with bispecific antibodies or CAR-T cells.

Ultimately, integrating TIL profiling into multi-modal diagnostic pipelines could facilitate a more personalized immuno-oncology approach in GBM with tailored immunotherapy design to the unique immune context of GBM.

### 4.5. Types of T Cell Dysfunction

GBM employs a multifaceted immunosuppressive strategy that primarily targets the effector functions of T cells, undermining effective antitumor immunity. As a result, T cells within the GBM tumor microenvironment exhibit several distinct forms of dysfunction, including senescence, tolerance, anergy, and exhaustion [85]. These states reflect impaired activation, proliferative capacity, and cytotoxic function, collectively contributing to the failure of immune surveillance and resistance to immunotherapeutic interventions.

Senescence is characterized by a hypofunctional state resulting from shortened telomeres. In human CD4^+^ and CD8^+^ T cells, telomere shortening appears to be the consequence of T-cell stimulation due to chronic inflammatory states, as is the case in cancer and GBM [107]. Phenotypically, senescent T cells are marked by the expression of CD57, a well-established indicator of terminal differentiation [108], along with the loss of key costimulatory molecules CD27 and CD28 [109]. In the context of GBM, CD4^+^ T cell immunosenescence has been associated with poor clinical outcomes: patients with elevated levels of CD4^+^CD28^−^CD57^+^ T cells exhibit significantly reduced overall survival [110]. Despite these associations, the molecular mechanisms involving telomere dynamics and telomerase activity in tumor-infiltrating T cells remain inadequately characterized in GBM and warrant further investigation.

Immune tolerance is a physiological mechanism that prevents autoimmunity by inducing T-cell unresponsiveness to self-antigens. In malignancies such as GBM, this mechanism is hijacked to suppress antitumor immunity. Tolerance is generally classified into two distinct processes: central and peripheral. Central tolerance occurs during T-cell development in the thymus, where T cells bearing T-cell receptors (TCRs) with excessively high affinity for self-antigen–MHC complexes undergo negative selection and are eliminated [111]. However, this process is not comprehensive. T cells reactive to tissue-restricted or tumor-associated antigens—often absent from thymic presentation—can escape deletion and enter the peripheral circulation. These escaped self-reactive T cells are then subjected to peripheral tolerance mechanisms, which act to restrain their activation and expansion [112]. GBM exploits peripheral tolerance to evade immune surveillance through multiple strategies. These include the induction of T-cell apoptosis via FasL-mediated deletion of infiltrating lymphocytes [113] and the expansion of Tregs [87,88], which further suppresses effector T-cell activity and reinforces local immune tolerance.

Exhaustion is defined as a progressive decrease in effector function, sustained expression of inhibitory receptors, loss of cytokine production, metabolic dysfunction, and distinct epigenetic and transcriptional alterations. Exhausted T cells are a distinct T-cell lineage defined by the progressive loss of proliferative capacity and secretory effector cytokine production [114]. In vitro and in vivo CRISPR-Cas9 screen on T cell exhaustion assay identified cBAF complex and, in particular, Arid1a expression as a critical step for the acquisition of exhaustion phenotype [115]. Although the delineation between T cell exhaustion and general dysfunction is not entirely clear, it is becoming evident that T cell exhaustion is a distinct cellular process accompanied by specific metabolic, transcriptional, and epigenetic changes. The complexity of understanding T cell exhaustion phenotype led to the division of these cells into progenitor exhausted T (Tex_prog) cells and terminally exhausted T (Tex_term) cells [116,117]. The Tex_prog represents a more stem/progenitor cell that conserves a lot of plasticity and can be identified by the expression of PD1 and TCF1. In opposition, the Tex_terms are short-lived with a strong antitumor function and are characterized by the high expression of TOX, PD1, and TIM3. Over time in GBM, the Tex_prog gets lost, and a subset of T cells acquires a more terminal exhaustion phenotype due to the presence of TAMs [66].

### 4.6. Consequences of T Cell Exhaustion in GBM

T cell exhaustion and dysfunction in GBM contribute to a range of immunological deficits that impair effective antitumor responses. One of the earliest hallmarks of T cell exhaustion is the loss of IL-2 production, a cytokine critical for initiating immune responses and promoting T-cell proliferation [118]. This is typically followed by a sequential decline in the production of other key effector molecules, including TNF-α, which mediates inflammatory signaling; IFN-γ, a cytokine essential for both innate and adaptive immune activation; and granzyme B, a serine protease required for the cytolytic function of cytotoxic T cells [119]. Exhausted T cells also exhibit sustained expression of multiple inhibitory receptors, such as programmed cell death protein 1 (PD-1), cytotoxic T-lymphocyte–associated protein 4 (CTLA-4), T-cell immunoglobulin and mucin domain–containing protein 3 (TIM-3), lymphocyte activation gene-3 (LAG-3), and T cell immunoreceptor with Ig and ITIM domains (TIGIT). These receptors serve as both markers and mediators of exhaustion by interacting with ligands in the tumor microenvironment, leading to suppressed T-cell activation, proliferation, and effector function. Several of these have been explored as potential immune checkpoint inhibitors. Antagonizing or blocking PD-1 and CTLA-4 are well-recognized FDA-approved anticancer strategies aimed at improving T-cell function in multiple malignancies, including melanoma and non-small-cell lung carcinoma; however, these strategies have shown only limited efficacy in GBM [120,121].

### 4.7. HLA Mutations and Ligandome

One of the major escape routes for the glioma cells to avoid T cell recognition is the downregulation of major histocompatibility complex (MHC), impairing antigen presentation [83]. A growing body of evidence supports the hypothesis that somatic mutations in the human leukocyte antigen (HLA) molecules contribute to the emergence of immune escape variants in GBM. HLA class I molecules are essential for presenting tumor-derived antigenic peptides to effector T cells, and their complete loss can enable tumors to evade adaptive immune surveillance [122,123,124]. A comparative HLA peptidome profiling against normal tissue to identify GBM-associated HLA ligand was performed using mass spectrometry, leading to the discovery of new targets that could be used for immunotherapy [125]. Additionally, deep sequencing to new HLA loci and to genes associated with antigen presentation, such as B2M, TAP1/2, and MICA/B, shows how somatic mutations in TAP1 and B2M could interfere with antigen presentation machinery [126]. Mutations and polymorphisms in HLA genes may help define distinct molecular subtypes of GBM with differing immune profiles. Notably, cancers harboring recurrent somatic HLA mutations have been found to display increased expression of cytolytic activity signatures, indicative of immune infiltration by effector lymphocytes, suggesting that altered HLA function may enable tumors to resist immune attack even in the presence of an active immune response, highlighting HLA disruption as a key mechanism of immune evasion in GBM [124]. Importantly, an increase in such mutations could be found in recurrent GBM [126].

The complex immunosuppressive landscape, which encompasses dysfunctional effector T cells, elevated Treg population, immune checkpoint effects, and lack of antigen presentation, explains why GBM remains largely refractory to immunotherapeutic strategies that are effective in other cancers.

The immunosuppressive nature of the GBM TME, shaped in large part by the abundance of TAMs and paucity of functional T cells within a heterogeneous tumor architecture of highly plastic tumor cells, creates a hostile setting for effective antitumor immunity. Within this landscape, T cells represent a critical but highly constrained component of the immune response. As detailed above, CD8^+^ cytotoxic T cells, CD4^+^ effector T cells, and regulatory T cells each contribute uniquely to the immune dynamics in GBM, with their clinical relevance increasingly linked to their functional state and spatial distribution. However, widespread T cell dysfunction, ranging from exhaustion and suppressive signaling to impaired antigen recognition due to HLA mutations and T-cell receptor clonal heterogeneity, limits their therapeutic potential. Understanding these layered mechanisms of immune escape is essential for guiding the development of targeted immunotherapies. In the following section, we explore how this foundational knowledge is being leveraged to design next-generation T cell-based strategies aimed at restoring effective immune surveillance and overcoming the barriers imposed by the GBM microenvironment.

## 5. The Future of T Cell-Based Strategies in GBM

GBM remains one of the most formidable challenges in oncology, but advances in T cell-based immunotherapy, particularly CAR T-cell therapy, offer a promising path forward. As research continues to evolve, several strategies are emerging to overcome the current limitations and optimize the clinical impact of T cell therapies in GBM.

A major barrier to effective CAR T-cell treatment in GBM is the blood–brain barrier, which impedes the trafficking of peripherally infused cells into the tumor site. To address this, strategies aimed at enhancing CAR-T cell delivery to the tumor site are being actively explored. One such approach involves direct intracranial infusion of CAR-T cells, bypassing the systemic circulation and the BBB altogether. A study by Brown et al. demonstrated the feasibility of this approach using IL13Rα2-targeted CD8^+^ T cells to treat recurrent GBM, laying the groundwork for a new generation of locoregional delivery strategies [127]. In parallel, ongoing efforts to modulate the tumor microenvironment aim to enhance T cell infiltration and persistence, making delivery strategies even more effective.

Identifying optimal tumor-associated antigens remains another critical step in enhancing the efficacy of CAR T-cell therapy for GBM, particularly given the disease’s profound intratumoral heterogeneity and antigen loss. Consequently, efforts have focused on identifying antigens that are not only highly expressed in GBM but also consistently present across the heterogeneous nature of GBM. A number of targets, namely EGFRvIII, CD70, CD133, B7H3, and IL13Rα2, are subjects of ongoing investigation through clinical trials [128]. Among these, IL13Rα2 is one of the most extensively studied, owing to its elevated expression in GBM cells and limited expression in normal brain tissue, making it a particularly promising candidate [129].

In tandem with increasing the number of potential targets for the CAR receptor, a strategy of combining CAR T cells with T cell-Engaging Antibody Molecules (TEAMs) presents a promising approach to the problem of antigen loss [130]. TEAMs are antibodies that link two single-chain variable fragments together, where one binds to the target antigen and the other binds to the T cell CD3 receptor. This combines antigen specificity with the ability to induce cytotoxicity in nearby bystander T cells. Several clinical trials have tried adopting this approach. In one study, EGFRvIII-targeting CAR T cells have been engineered to secrete TEAMs against wild-type EGFR, as antigen loss in tumors treated with EGFRvIII CAR alone is correlated with tumor persistence and amplification of wild-type EGFR [131]. These combinatorial approaches aim to expand the breadth of tumor recognition, mitigate immune escape due to antigen loss, and enhance the overall durability of CAR T-cell responses.

Although clinical trials using checkpoint inhibitors in GBM have shown limited efficacy as monotherapies, combining checkpoint blockade with CAR T-cell therapy holds promise for enhancing antitumor responses. Preclinical studies support this approach; for instance, co-inhibition of immune checkpoints such as PD-1, CTLA-4, and TIM-3 alongside CAR T-cell therapy has demonstrated improved tumor control and prolonged survival in the murine and canine models [132]. Notably, the efficacy of checkpoint blockade appears to depend on the specific CAR T-cell construct employed, as CAR T cells targeting EGFRvIII and IL13Rα2 induce distinct checkpoint expression profiles within the tumor microenvironment [133]. However, clinical translation remains challenging. A recent phase I trial combining EGFRvIII-targeted CAR T cells with PD-1 inhibition failed to show significant clinical benefit, underscoring the complexity of these interactions [134]. Continued research will be critical to identifying optimal CAR T/checkpoint inhibitor pairings, refining timing and dosing strategies, and uncovering biomarkers to guide patient selection.

As the field advances, the integration of multi-targeted strategies with a nuanced understanding of GBM’s molecular and genomic diversity will be critical to overcome tumor heterogeneity. Equally important is unraveling the complexities of the immunosuppressive microenvironment and resistance mechanisms that hinder durable responses. These insights will inform the rational design of next-generation T-cell therapies that are both more precise and more effective. With continued innovation, T cell-based immunotherapies are poised to become a transformative and personalized approach in the fight against this challenging disease.

## 6. Conclusions

Intratumoral heterogeneity in glioblastoma arises not only from distinct genomic alterations but also from the remarkable plasticity of glioma cells and their ability to adopt diverse cellular states depending on their anatomical location. These cells can reactivate neurodevelopmental programs, mimicking oligodendrocyte or neural progenitor behavior, and exploit neuronal networks for their own advantage. The tumor microenvironment, particularly the hypoxic niche, plays a key role in driving clonal evolution, with each clone adapting to and reshaping its surroundings to promote survival and expansion. As this process unfolds, immune cell function, including that of T cells and myeloid cells, becomes progressively impaired, fostering an immunosuppressive microenvironment. This immunosuppressive tumor microenvironment represents a major obstacle to effective antitumor immunity and a central reason for the limited success of current immunotherapeutic approaches in GBM. Despite growing insights into individual components of the TME, our understanding of how immune dysfunction evolves in relation to tumor cell heterogeneity remains incomplete. Key knowledge gaps include the mechanisms governing T cell exclusion and exhaustion, the dynamic interplay between glioma cells’ heterogeneity and immune suppression, and the impact of regional microenvironmental cues, such as hypoxia, metabolic stress, and neuronal signaling, on immune cell fate and function.

Addressing these gaps will require integrative, high-resolution mapping of tumor and immune cell states across space and time, as well as functional validation in clinically relevant models. Ultimately, a deeper and more systems-level understanding of the cellular and molecular components of the GBM microenvironment is essential to inform the rational design of immunotherapies and combinatorial strategies that can overcome resistance, reawaken antitumor immunity, and improve patient outcomes.

## Data Availability

No new data were created or analyzed in this study.
